# Advisory Committee on Immunization Practices Recommended Immunization Schedule for Children and Adolescents Aged 18 Years or Younger — United States, 2025

**DOI:** 10.15585/mmwr.mm7402a2

**Published:** 2025-01-16

**Authors:** Anindita N. Issa, A. Patricia Wodi, Charlotte A. Moser, Sybil Cineas

**Affiliations:** ^1^Immunization Services Division, National Center for Immunization and Respiratory Diseases, CDC; ^2^Vaccine Education Center, Children's Hospital of Philadelphia, Philadelphia, Pennsylvania; ^3^The Warren Alpert Medical School of Brown University, Providence, Rhode Island.

At its October 2024 meeting, the Advisory Committee on Immunization Practices* (ACIP) approved the Recommended Immunization Schedule for Child and Adolescent Ages 18 Years or Younger, United States, 2025. The schedule supports health care providers, as well as public health and other professionals, by providing a consolidated summary of current ACIP recommendations for vaccinating children and adolescents. The 2025 schedule includes several updates to the cover page, tables, notes, and appendix.^†^ The addendum remains part of the schedule and will be used to summarize new or updated ACIP recommendations that occur before the next annual schedule update. Health care providers are strongly encouraged to use all parts of the schedule (the cover page, tables, notes, appendix, and addendum) together when making recommendations for individual patients. The 2025 child and adolescent immunization schedule can be found on the CDC website (https://www.cdc.gov/vaccines/hcp/imz-schedules/index.html).

Consistent with previous years’ schedules, the 2025 child and adolescent immunization schedule is recommended by ACIP (https://www.cdc.gov/acip/index.html) and approved by CDC (https://www.cdc.gov), the American Academy of Pediatrics (https://www.aap.org), the American Academy of Family Physicians (https://www.aafp.org/home.html), the American College of Obstetricians and Gynecologists (https://www.acog.org/), the American College of Nurse-Midwives (https://www.midwife.org), the American Academy of Physician Associates (https://www.aapa.org), and the National Association of Pediatric Nurse Practitioners (https://www.napnap.org).

ACIP’s recommendations for use of each vaccine and other immunizing agents are developed after in-depth reviews of current product-related data, including the epidemiology and societal impacts of the vaccine-preventable disease; efficacy, effectiveness, and safety of the vaccine or other immunizing agent; quality of evidence; feasibility of program implementation; impact on health equity; and economic analyses of immunization policy (*1*,*2*). For each vaccine in the schedule, clinical trials are conducted in the context of standard-of-care related to the routine childhood immunization schedule (*3*). Routinely recommended vaccines are monitored by CDC and the Food and Drug Administration (FDA) for safety through ongoing and cumulative efforts, including multiple surveillance systems, safety studies, and review of the literature (https://www.cdc.gov/vaccine-safety-systems/about/cdc-monitoring-program.html). Recommendations for specific vaccines and related agents that occur between annual schedule updates^§^ will be summarized in the addendum section; however, health care providers should refer to detailed ACIP recommendations for use of each product (https://www.cdc.gov/acip-recs/hcp/vaccine-specific/index.html). ACIP vaccine recommendations do not establish mandates.

The use of trade names in this report and in the child and adolescent immunization schedule is for identification purposes only and does not imply endorsement of a specific product by ACIP or CDC.

## Changes in the 2025 Child and Adolescent Immunization Schedule

Compared with the 2024 child and adolescent schedule, changes in the 2025 immunization schedule for children and adolescents include new and updated recommendations for COVID-19 vaccines (*4*), *Haemophilus influenzae* type b vaccines (Hib) (*5*), influenza vaccines (*6*), and meningococcal serogroup B vaccines (*7*). In all sections of the schedule, recommended influenza vaccines have been changed from the quadrivalent to trivalent formulation to be consistent with the vaccine products approved by FDA for the 2024–25 influenza season.

Other changes include clarification of recommendations for dengue vaccine; diphtheria and tetanus toxoids and acellular pertussis vaccines (DTaP); inactivated poliovirus vaccine (IPV); measles, mumps, and rubella virus vaccines (MMR); measles, mumps, rubella, and varicella virus vaccines (MMRV); pneumococcal vaccines; respiratory syncytial virus monoclonal antibody (RSV-mAb); respiratory syncytial virus vaccines (RSV); and varicella vaccine (VAR).

### Cover Page

• Trivalent cell culture–based inactivated influenza vaccine was added to the table listing abbreviations and trade names of vaccines and other immunizing agents.

### Table 1 (Age-Based Immunization Schedule)

• **COVID-19 row:** The text overlay was revised to reflect updated vaccination recommendations. This text overlay now states, “1 or more doses of 2024–2025 vaccine (See Notes).”

• **Dengue row:** For children and adolescents aged 9–16 years, the color was changed to purple, clarifying that vaccination is recommended for some children and adolescents in this age group. In addition, the definition of the purple box in the legend was revised so the text reads, “Range of recommended ages for certain high-risk groups or populations.”

• **Influenza rows:** The text overlay was revised to harmonize with the adult schedule and now states, “1 or 2 doses annually” or “1 dose annually.”

• **IPV row:** The column for age 18 years was changed from gray to green, indicating catch-up vaccination is recommended. In addition, the text “<18 years” was deleted from the vaccine column.

• **Legend:** The definition of the gray box in the legend was revised to harmonize with the definition in Table 3. The definition now states, “No Guidance/Not Applicable.”

### Table 2 (Catch-Up Immunization Schedule)

• There were no revisions to Table 2.

### Table 3 (Immunization Schedule by Medical Indication)

• **COVID-19 row:** In the columns for children and adolescents who are immunocompromised (excluding HIV infection) and for those with HIV infection and CD4+ T-lymphocyte count <15% or <200/mm^3^, the yellow bar was changed to brown to reflect that additional doses are recommended.

• **Influenza (inactivated) row:** A text overlay was added to the column for children and adolescents who are immunocompromised (excluding HIV infection). The text overlay now states, “Solid organ transplant: 18 years (See Notes),” directing health care providers to review the influenza vaccination notes because there is a recommendation for adding trivalent high-dose inactivated influenza vaccine (HD-IIV3) and trivalent adjuvanted inactivated influenza vaccine (aIIV3) to the vaccines that may be administered to solid organ transplant recipients aged 18 years who are receiving immunosuppressive medications.

### Vaccine Notes

The notes for each vaccine and related agent are presented in alphabetical order. Edits have been made throughout the Notes section to harmonize language, to the greatest extent possible, with language in the adult immunization schedule.

• **COVID-19:** The “Routine vaccination” and “Special situations” sections were revised to reflect recommendations for use of 2024–2025 COVID-19 vaccine in children and adolescents. The “Routine vaccination” section describes recommendations for the general population, and the “Special situations” section describes recommendations for persons who are moderately or severely immunocompromised. In each section, the recommendations are outlined by age group and previous COVID-19 vaccination history. In addition, hyperlinks to the interim clinical considerations for use of COVID-19 vaccines as well as Emergency Use Authorization indications for COVID-19 vaccines are included.

• **DTaP:** The “Special situations” section now includes a summary of guidance for use of tetanus and diphtheria vaccine (Td) in children aged <7 years who have a contraindication specific to the pertussis component of DTaP.

• **Hib:** In the “Routine vaccination” section, Vaxelis was added as a second preferred option for primary doses in American Indian and Alaska Native infants. In the “Special situations” section, early component complement inhibitor use was added as an indication for vaccination if age appropriate.

• **Hepatitis B:** Language regarding vaccines not recommended for use during pregnancy was revised to remove Heplisav-B.

• **Influenza:** Language was added to the “Routine vaccination” section stating that persons aged 18 years who are solid organ transplant recipients receiving immunosuppressive medications may receive HD-IIV3 or aIIV3 without preference over other age-appropriate trivalent inactivated or recombinant influenza vaccines.

• **MMR:** In the “Special situations” section, the recommendation for international travel was revised for clarity, described both by age group and MMR vaccination history.

• **Meningococcal serogroup B:** The “Routine vaccination” and “Special situations” sections were revised to include the new Bexsero vaccination schedule. For healthy persons aged 16–23 years, a series of 2 doses separated by 6 months is recommended, based on shared clinical decision-making. Children and adolescents aged ≥10 years at increased risk for serogroup B meningococcal disease are recommended to receive a 3-dose series at 0-, 1–2-, and 6-month intervals.

• **Pneumococcal:** Language was added to clarify that, because of limited data, there is no recommendation for use of pneumococcal conjugate vaccines or 23-valent pneumococcal polysaccharide vaccine during pregnancy.

• **RSV-mAb:** The “Routine vaccination” section was revised to state that infants born during October–March should be immunized within 1 week of birth, ideally during the birth hospitalization. Information was added to clarify that infants born to mothers who received RSV vaccination during a previous pregnancy should receive nirsevimab. In addition, a revision was made to clarify that for infants born during April–September, the optimal time of year to administer RSV-mAb is October–November.

• **RSV:** The “Routine vaccination” section was revised to clarify that additional doses are not recommended in subsequent pregnancies.

### Appendix (Contraindications and Precautions)

• **Hepatitis B row:** In the “Contraindicated and Not Recommended” column, the language about vaccines not recommended for use during pregnancy was revised to remove Heplisav-B. The corresponding footnote with hyperlink to the pregnancy registries was also revised to remove information for the Heplisav-B registry, which is no longer active.

• **MMR/MMRV row:** In the “Contraindicated and Not Recommended” column, information stating that use of MMRV is contraindicated in persons with HIV infection of any severity was added. In addition, language was added to the “Precautions” column directing health care providers to review the Varicella/MMRV row if using MMRV.

• **Varicella row:** In the “Contraindicated and Not Recommended” column, information stating that use of MMRV is contraindicated in persons with HIV infection of any severity was added. In addition, the name for the Varicella row has been changed to “Varicella/MMRV.”

### Additional Information

The Recommended Child and Adolescent Immunization Schedule, United States, 2025, is available at https://www.cdc.gov/vaccines/hcp/imz-schedules/child-adolescent-age.html. The full ACIP recommendations for each vaccine are also available at https://www.cdc.gov/acip-recs/hcp/vaccine-specific/index.html. All vaccines and immunizing agents identified in Tables 1, 2, and 3 (except dengue, DTaP, rotavirus, MMRV, and nirsevimab) also appear in the Recommended Adult Immunization Schedule for Ages 19 Years or Older, United States, 2025, available at https://www.cdc.gov/vaccines/hcp/imz-schedules/adult-age.html. The notes and appendix for vaccines that appear in both the child and adolescent immunization schedule and the adult immunization schedule have been harmonized to the greatest extent possible.

## Footnotes

* Recommendations for routine immunization of children and adolescents are developed by ACIP, a federal advisory committee chartered to provide expert external advice and guidance to the CDC director on use of vaccines and related agents for the control of vaccine-preventable diseases in the civilian population of the United States. Recommendations for routine immunization of children and adolescents are harmonized to the greatest extent possible with recommendations made by the American Academy of Pediatrics, the American Academy of Family Physicians, the American College of Obstetricians and Gynecologists, the American College of Nurse-Midwives, the American Academy of Physician Associates, and the National Association of Pediatric Nurse Practitioners. ACIP recommendations become official agency guidelines once the recommendation has been adopted by the CDC director. Additional information about ACIP is available at https://www.cdc.gov/acip/index.html.

† Past immunization schedules are available at https://www.cdc.gov/vaccines/hcp/imz-schedules/resources.html.

§ CDC encourages organizations to use syndication as a more reliable method for displaying the most current and accurate immunization schedules on an organization’s website rather than copying these schedules to their websites. Use of content syndication requires a one-time step that ensures an organization’s website displays current schedules as soon as they are published or revised; instructions for syndication code are available on CDC’s website (https://www.cdc.gov/vaccines/hcp/imz-schedules/syndicate-resources.html). CDC also offers technical assistance for implementing this form of content syndication (requests can be emailed to ncirdwebteam@cdc.gov).
